# HRD1 prevents apoptosis in renal tubular epithelial cells by mediating eIF2α ubiquitylation and degradation

**DOI:** 10.1038/s41419-017-0002-y

**Published:** 2017-12-11

**Authors:** Yujie Huang, Yifei Sun, Yizhi Cao, Hui Sun, Min Li, Hui You, Dongming Su, Yanjiao Li, Xiubin Liang

**Affiliations:** 1 Renal Division, Sir Run Run Hospital, Nanjing Medical University, Nanjing, 211166 Jiangsu China; 2 Department of Pathology, Jiangsu Province Hospital of TCM, Affiliated Hospital of Nanjing University of TCM, Nanjing, 210026 Jiangsu Province China; 3 Department of Pathophysiology, Nanjing Medical University, Nanjing, 211166 Jiangsu China; 4 Center of Pathology and Clinical Laboratory, Sir Run Run Hospital, Nanjing Medical University, Nanjing, 211166 Jiangsu Province China; 5 Department of Endocrinology, The Second Hospital of Shanxi Medical University, Taiyuan, 030001 Shanxi Province China

## Abstract

Apoptosis of renal tubular epithelial cells is a key feature of the pathogenicity associated with tubulointerstitial fibrosis and other kidney diseases. One factor that regulates important cellular processes like apoptosis and cell proliferation is HRD1, an E3 ubiquitin ligase that acts by promoting ubiquitylation and degradation of its target protein. However, the detailed mechanisms by which HRD1 acts as a regulator of apoptosis in renal tubular epithelial cells have not been established. In our previous liquid chromatography-tandem mass spectrometry (LC-MS/MS) study (Mol Endocrinol. 2016;30:600–613), we demonstrated that one substrate of HRD1 was eIF2α, a critical protein in the PERK-eIF2α-ATF4-CHOP signaling pathway of endoplasmic reticulum (ER) stress. Here, we show that eIF2α expression was increased and HRD1 expression decreased when apoptosis was induced in HKC-8 cells by palmitic acid (PA) or high glucose (HG). HRD1 expression was also lower in kidney tissues from mice with diabetic nephropathy (DN) than in control mice. Forced expression of HRD1 also inhibited apoptosis in HKC-8 cells, while HRD1 overexpression decreased the expression of phosphorylated eIF2α and eIF2α. Further analysis indicated that HRD1 interacted with eIF2α and promoted its ubiquitylation and degradation by the proteasome. Moreover, the HRD1 protection of PA-treated HKC-8 cells was blunted by transfection with Myc-eIF2α. Thus, eIF2α ubiquitylation by HRD1 protects tubular epithelial cells from apoptosis caused by HG and PA, indicating a novel upstream target for therapeutic prevention of renal tubulointerstitial injury.

## Introduction

Renal tubular epithelial cells are the primary targets of a variety of kidney injury regardless of the initial insults. Renal tubular atrophy is often characterized in the histopathological staining of the kidney lesions of patients with chronic kidney disease (CKD)^1^. Injured tubular cells present the consequence phenomenon of cell proliferation, apoptosis, autophagy, and the endothelial–mesenchymal transition.

Accumulating evidence now indicates that the apoptosis of tubular epithelial cells is a crucial step in the pathogenesis of progressive tubulointerstitial fibrosis^2,3^. For this reason, treatments that can decrease apoptosis, such as addition of bone morphogenetic protein-7^4^ or an angiotensin receptor blocker^5^, are beneficial and can prevent the progression of fibrosis. Previous work has shown that high-glucose levels (HG) are an initiating factor that promotes the generation of reactive oxygen species and subsequent apoptosis in tubular epithelial cells. This apoptosis induced by HG has been verified by the morphological changes observed during the development of diabetic nephropathy (DN)^6^. Abnormal lipid metabolism and lipotoxicity also accelerate the progression of renal injury^7,8^. Lipid disorders induce renal oxidative stress, endoplasmic reticulum (ER) stress, and inflammatory processes in podocytes, mesangial cells, and tubular epithelial cells^9,10^. Many studies have reported the occurrence of renal cell apoptosis in response to treatment with palmitic acid (PA)^10,11^. However, the molecular mechanisms underlying tubular epithelial cell apoptosis remain unclear.

ER stress, which is caused by the presence of unfolded or misfolded proteins, has been linked to various kidney diseases, including DN, renal fibrosis, and acute kidney injury^12,13^. In the ER, secretory and membrane proteins, if unfolded or misfolded, can be identified by ER chaperones and degraded by the ER-associated degradation (ERAD) machinery^14^. The accumulation of misfolded or unfolded proteins in the ER will trigger ER stress-mediated apoptosis by the unfolded protein response (UPR)^15^. This response in mammalian cells activates three signaling pathways: the PERK-eIF2α-ATF4-CHOP, IRE1-TRAF2-ASK1, and ATF6 pathways^16^. The IRE1 and ATF6 pathways increase the expression of ERAD components and ER chaperones, respectively, whereas activation of protein kinase-like endoplasmic reticulum kinase (PERK) recruits and phosphorylates its substrate, eukaryotic initiation factor (eIF2α). The phosphorylated eIF2α (p-eIF2α) then inhibits and reduces general protein translation in cells, while paradoxically activating the translation of activating transcription factor 4 (ATF4) mRNA, a key transducer. Consequently, the transcription of C/EBP homologous protein (CHOP) after translocation of ATF4 into the nucleus decreases Bcl-2 expression and ultimately leads to the apoptosis seen in response to ER stress^17,18^.

Activation of the PERK-eIF2α-ATF4 pathway confirms that eIF2α plays an important role in ER stress-induced apoptosis. Our previous LC-MS/MS analysis of injured tubular epithelial cells (HKC-8) revealed that eIF2α was a substrate of 3-hydroxy-3-methylglutaryl reductase degradation protein (HRD1), an ERAD-associated E3 ubiquitin ligase^19^. HRD1 physically promotes the degradation of proteins in processes such as renal injury and obesity^19–21^. Taken together, these findings indicated that eIF2α could undergo ubiquitylation by HRD1, followed by further downregulation through ERAD. Whether this represents the mechanism regulating tubular epithelial cell apoptosis is unknown and is the focus of the present study.

Here, we confirm that HRD1 is downregulated and eIF2α increases in apoptotic tubular epithelial cells. Overexpression of HRD1 mediates eIF2α ubiquitylation and decreases eIF2α expression, resulting in amelioration of tubular epithelial cell apoptosis. This study demonstrates a new mechanism for tubular epithelial cell apoptosis and points to a new direction for the development of therapeutic strategies for renal injury.

## Results

### Glucose and PA induces apoptosis of HKC-8 cells

**Fig. 1:**
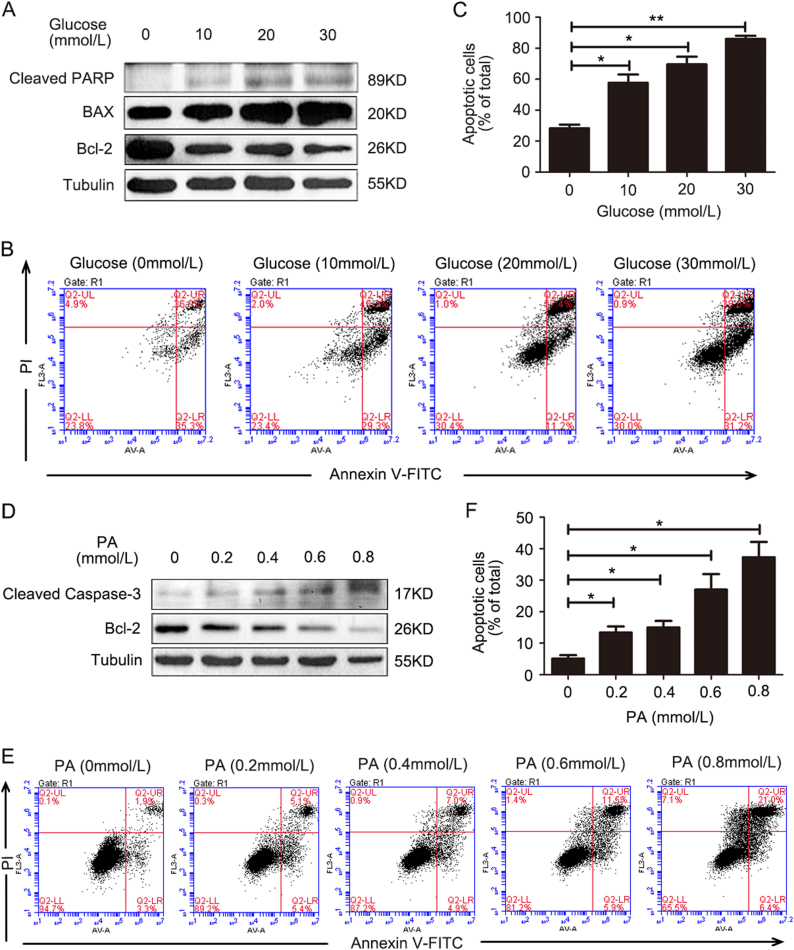
Glucose and palmitic acid induces apoptosis in HKC-8 cells. **a** HKC-8 cells were treated with glucose (0, 10, 20, and 30 mmol/l) for 24 h. The cleaved PARP and BAX expressions were dose-dependently increased, but Bcl-2 expression was decreased with high-glucose treatment. **b** Flow cytometry measurements showed that high-glucose treatment induced HKC-8 cell apoptosis. **c** The apoptotic cells in (**b**) were quantified. **d** HKC-8 cells were treated with palmitic acid (PA; 0, 0.2, 0.4, 0.6, and 0.8 mmol/l) as an injury factor for 24 h. The cleaved caspase-3 expression was increased, but Bcl-2 expression was decreased in a dose-dependent manner. **e** Flow cytometry measurements showed that PA treatment induced HKC-8 cell apoptosis. **f** The apoptotic cells in (**e**) were quantified. Bars are means ± S.E. from three independent experiments. *P *<* 0.05, **P *<* 0.01

HG levels and lipotoxicity are known critical injury factors for renal tubular epithelia. In our studies, HKC-8 cells were treated with glucose (0, 10, 20, and 30 mmol/l) for 24 h. The expression of cleaved PARP, Bcl-2, and BAX was then examined using western blotting. As shown in Fig. 1a, cleaved PARP and BAX expressions showed dose-dependent increases, but Bcl-2 expression was decreased with glucose treatment. In addition, glucose also induced dose-dependent apoptosis, as determined by the flow cytometry assay (Fig. 1b) and quantification of apoptotic cells (Fig. 1c). Exposure of HKC-8 cells to PA (0, 0.2, 0.4, 0.6, and 0.8 mmol/l) for 24 h as an injury treatment increased the expression of cleaved caspase-3, but PA dose-dependently decreased Bcl-2 expression (Fig. 1d). Flow cytometry measurements showed that PA treatment significantly induced HKC-8 cell apoptosis (Fig. 1e), as confirmed by the numbers of apoptotic cells (Fig. 1f). These data suggested that either HG or PA induced renal tubular epithelial cell apoptosis, in agreement with a previous report^6,11^. The quantification of dead cells in response to increased glucose or PA was shown in Supplementary Fig. 1.

### HRD1 expression is decreased in the kidneys of *db/db* mice

**Fig. 2:**
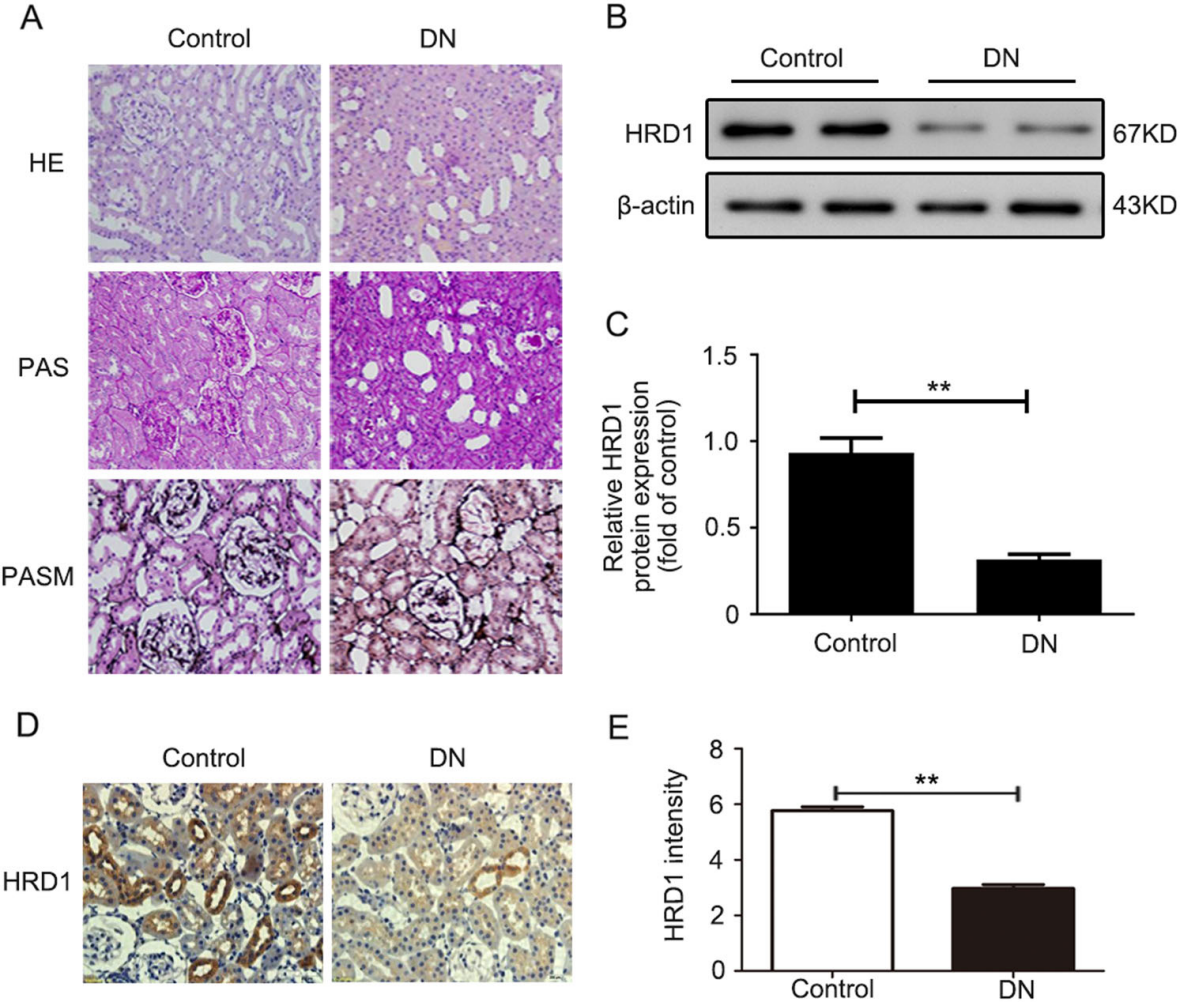
HRD1 expression is decreased in the kidneys of *db/db* mice. **a** Glomerulosclerosis and tubulointerstitial fibrosis were observed in a mouse model of diabetic nephropathy (DN; *db/db* mice) when compared with the control mice. **b** The HRD1 expression was tested using kidney tissues from *db/db* mice and control mice by western blot assays. **c** Quantitation of immunoblot data for HRD1 proteins as in (**b**). **d** The kidney expression of HRD1 was significantly decreased in the DN model as determined by immunohistochemical staining (×400). **e** The intensity of HRD1 expression was quantified. Bars are means ± S.E. from three independent experiments. **P < 0.01

The *db/db* mice were chosen to determine the expression of HRD1, an E3 ubiquitin ligase, in kidney tissues here because *db/db* mice develop detectable DN, whereas *db/m* mice served as the nondiabetic controls. Hematoxylin and eosin (HE), periodic acid-Schiff (PAS), and periodic Schiff-methenamine (PASM) staining were performed to assess morphological changes in the *db/db* mice. The results revealed marked glomerulosclerosis and tubulointerstitial fibrosis in the *db/db* mice compared with the control mice (Fig. 2a). The HRD1 expression was significantly decreased in kidney tissues of *db/db* mice vs. *db/m* mice, as evaluated by western blotting (Fig. 2b) and quantification of HRD1 expression (Fig. 2c), which is consistent with our previous work^19^. Meanwhile, in the present study, the decreased HRD1 protein in *db/db* mice was also verified by immunohistochemical staining of kidney tissues of *db/db* mice as shown in Fig. 2d, and IHC staining density of HRD1 expression was shown in Fig. 2e.

### HRD1 is downregulated in apoptotic HKC-8 cells

**Fig. 3:**
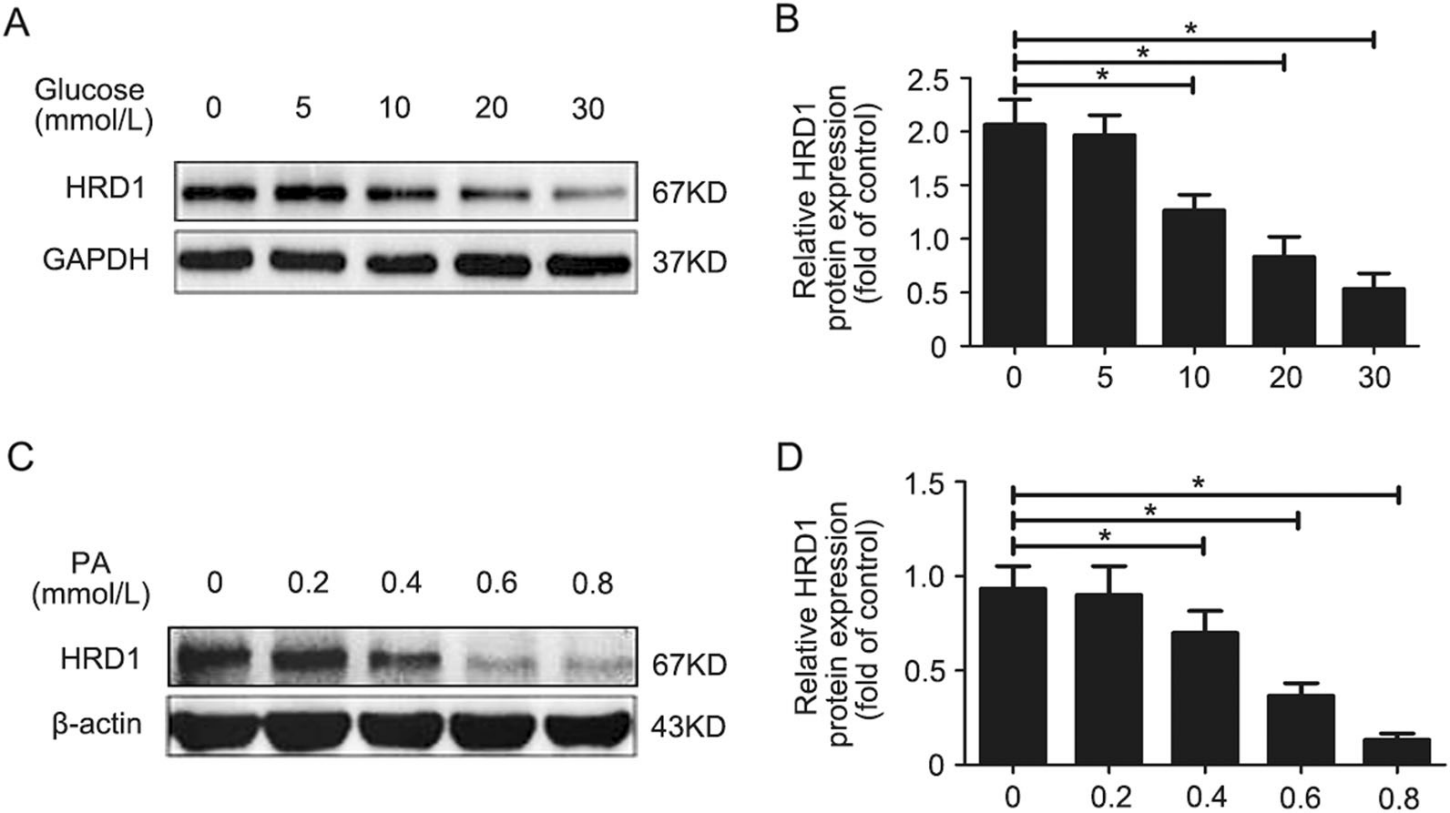
HRD1 is downregulated in apoptotic HKC-8 cells. **a** Glucose treatment inhibited HRD1 expression in a dose-dependent manner. **b** Quantification of HRD1 expression in (**a**), normalized to GAPDH expression. **c** Palmitic acid (PA) treatment also inhibited HRD1 expression in a dose-dependent manner. **d** Quantification of HRD1 expression in (**c**), normalized to actin expression. Bars are means ± S.E. from three independent experiments. *P *<* 0.05

HRD1 expression was determined by western blotting in HKC-8 cells treated with glucose (0, 5, 10, 20, and 30 mmol/l) for 24 h. The glucose treatment inhibited HRD1 expression in a dose-dependent manner (Fig. 3a and Supplementary Fig. 2). The means of HRD1 protein expression in HKC-8 cells treated with glucose are shown in Fig. 3b. The PA treatment (0, 0.2, 0.4, 0.6, and 0.8 mmol/l) resulted in a similar dose-dependent inhibition of HRD1 expression (Fig. 3c). The means of HRD1 expression in HKC-8 cells treated with PA are shown in Fig. 3d. These data demonstrated that HRD1 expression was downregulated in HKC-8 cells following the induction of apoptosis by either HG or PA.

### HRD1 overexpression prevents PA-induced apoptosis of HKC-8 cells

**Fig. 4:**
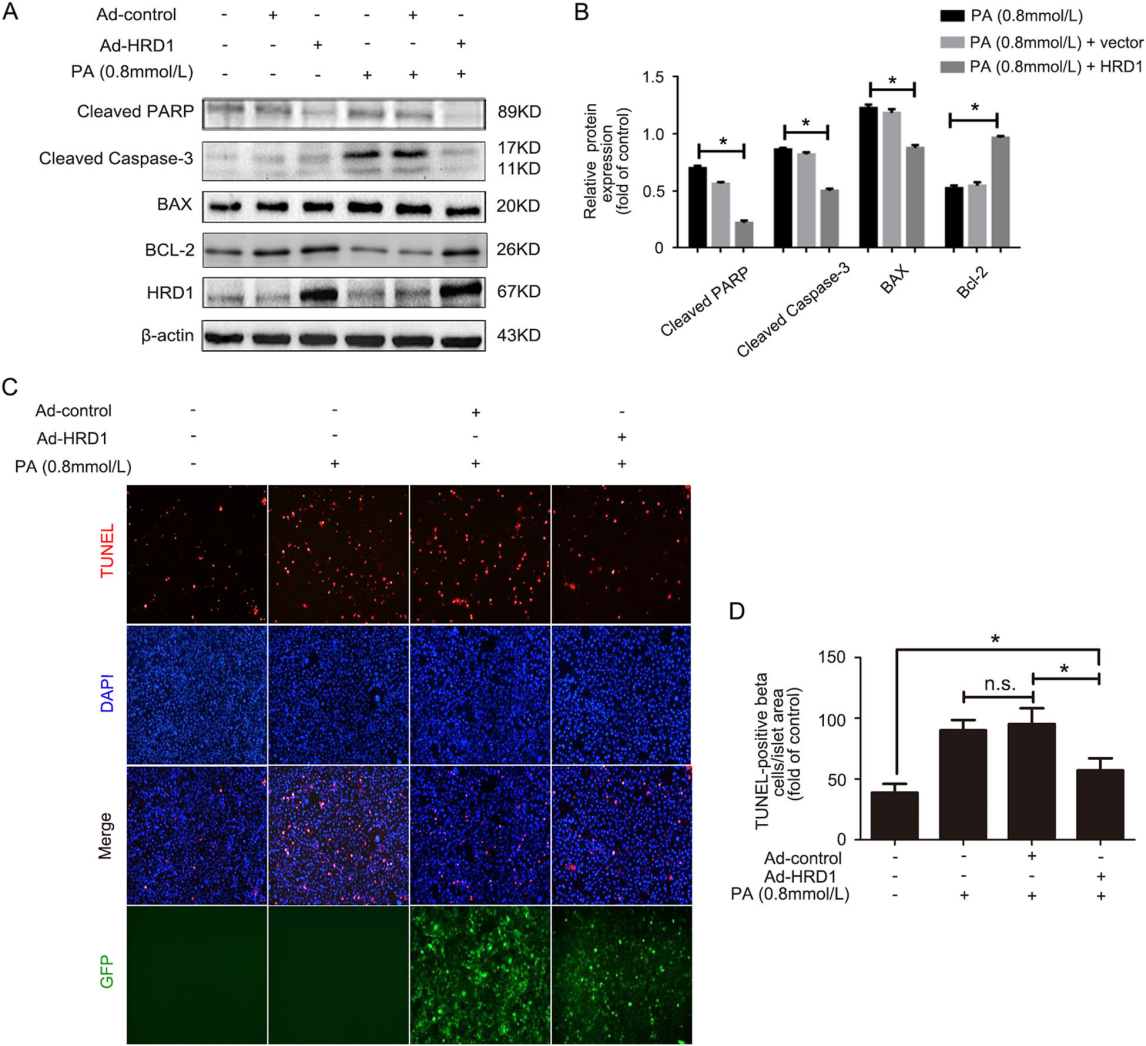
HRD1 overexpression prevents PA-induced apoptosis of HKC-8 cells. **a** Palmitic acid (PA) treatment significantly increased the expressions of cleaved PARP, cleaved caspase-3, and BAX, but decreased Bcl-2 expression. However, Ad-HRD1 infection suppressed the PA-induced expression of cleaved PARP, cleaved caspase-3, and BAX. **b** Quantitation of western blot data for protein expressions as in (**a**), normalized to actin expression. **c** TUNEL staining showed that Ad-HRD1 infection reduced the numbers of apoptotic HKC-8 cells compared to cells treated with PA. **d** The means of data of TUNEL-positive area as in (**c**). Bars are means ± S.E. from three independent experiments. n.s. means no significance, *P < 0.05

The role of HRD1 in PA-induced HKC-8 cell apoptosis was investigated in HKC-8 cells infected with Ad-HRD1 for 24 h, followed by treatment with or without PA (0.8 mmol/l) for 48 h. Western blots were performed to examine the apoptosis-related gene expression of cleaved PARP, cleaved caspase-3, BAX, and Bcl-2. PA significantly increased the expressions of cleaved PARP, cleaved caspase-3, and BAX, which are apoptosis-promoting genes, but decreased Bcl-2 expression. However, Ad-HRD1 infection suppressed the PA-induced expression of cleaved PARP, cleaved caspase-3, and BAX (Fig. 4a). The means for the expressions of these proteins are shown in Fig. 4b. The data indicated that overexpression of HRD1 prevented PA-induced HKC-8 cell apoptosis. Terminal dexynucleotidyl transferase-mediated dUTP nick end labeling (TUNEL) staining showed that Ad-HRD1 infection reduced the numbers of apoptotic HKC-8 cells seen with PA alone (Fig. 4c), thereby confirming the expression data. The means of data for the TUNEL-positive areas in HKC-8 cells are shown in Fig. 4d.

### HRD1 physically interacts with eIF2α

HRD1 is an E3 ubiquitin ligase that recognizes its specific substrate for ubiquitin-mediated degradation. In our previous LC-MS/MS study, we identified 54 peptides that could be candidate HRD1 substrates for ubiquitylation in HKC-8 cells. The top 10 candidate proteins included eIF2α as a sensitive substrate of HRD1^19^. Co-immunoprecipitation (Co-IP) analysis confirmed the physical interaction between HRD1 and eIF2α.

**Fig. 5:**
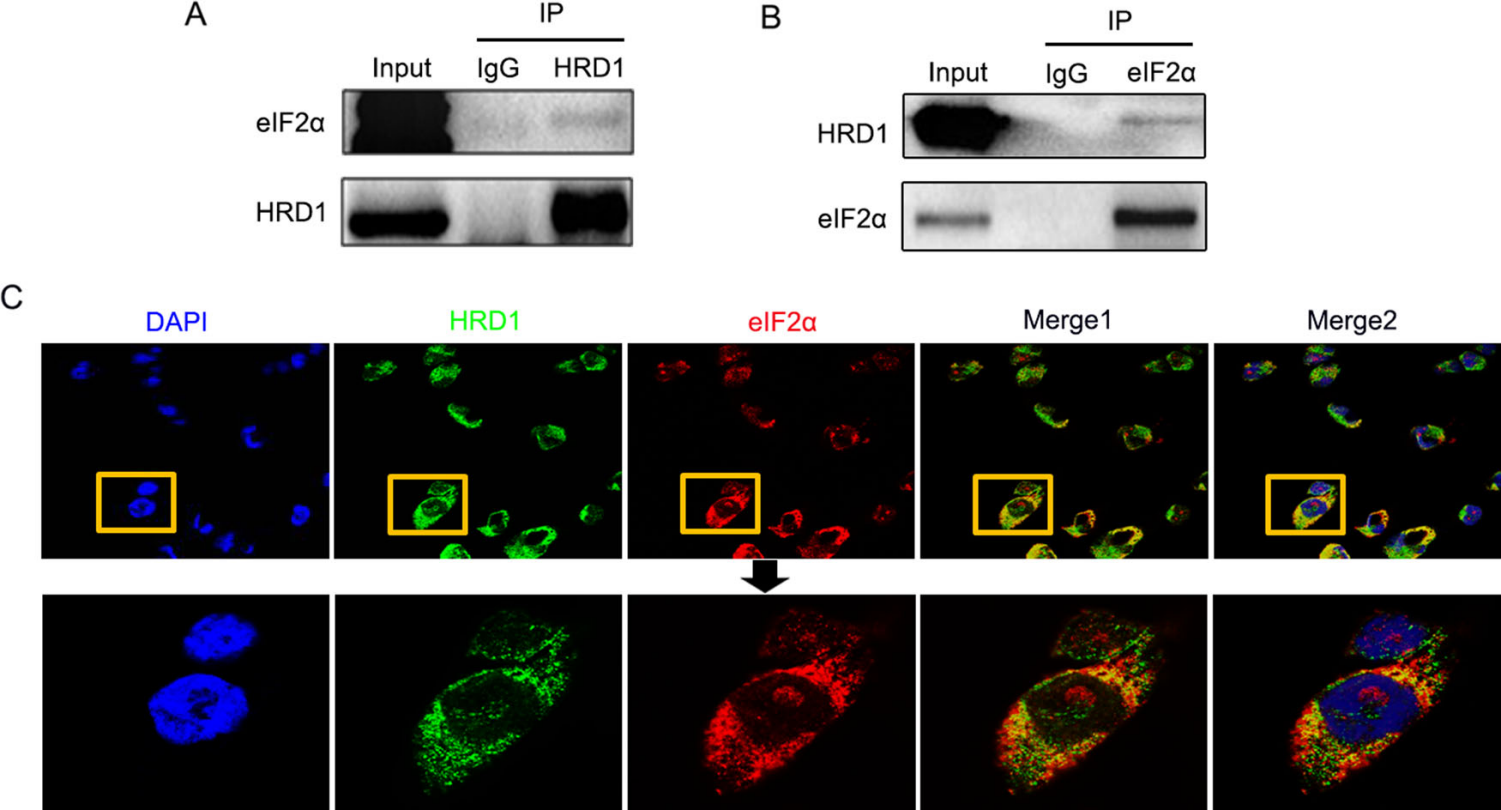
HRD1 physically interacts with eIF2α. **a** The interaction between HRD1 and eIF2α was detected in the co-IP analysis HKC-8 cells. **b** The binding signals of eIF2α with HRD1 were also confirmed using anti-HRD1 antibodies in IPs, and subsequent blotting with eIF2α antibodies. **c** The expressions of HRD1 and eIF2α were endogenous and intracytoplasmic. Merged images showed a significant colocalization

HRD1 antibodies were used to isolate protein complexes from HKC-8 cells, and these complexes were subsequently blotted with eIF2α antibodies. As shown in Fig. 5a, immunoprecipitations (IPs) performed with IgG as a control yielded no eIF2α signal, and a physical interaction between HRD1 and eIF2α was detected. The binding signals of eIF2α with HRD1 were also confirmed by IPs using anti-HRD1 antibodies and subsequent blotting with eIF2α antibodies (Fig. 5b). We further verified the interaction of HRD1 and eIF2α in HKC-8 cells by immunofluorescence staining using mouse anti-HRD1 and rabbit anti-eIF2α antibodies. As shown in Fig. 5c, the expressions of HRD1 and eIF2α were endogenous and intracytoplasmic. Merged images showed a significant colocalization, which was consistent with the expression data presented in Figs. 5a, b.

### HRD1 promotes eIF2α ubiquitylation and degradation

**Fig. 6:**
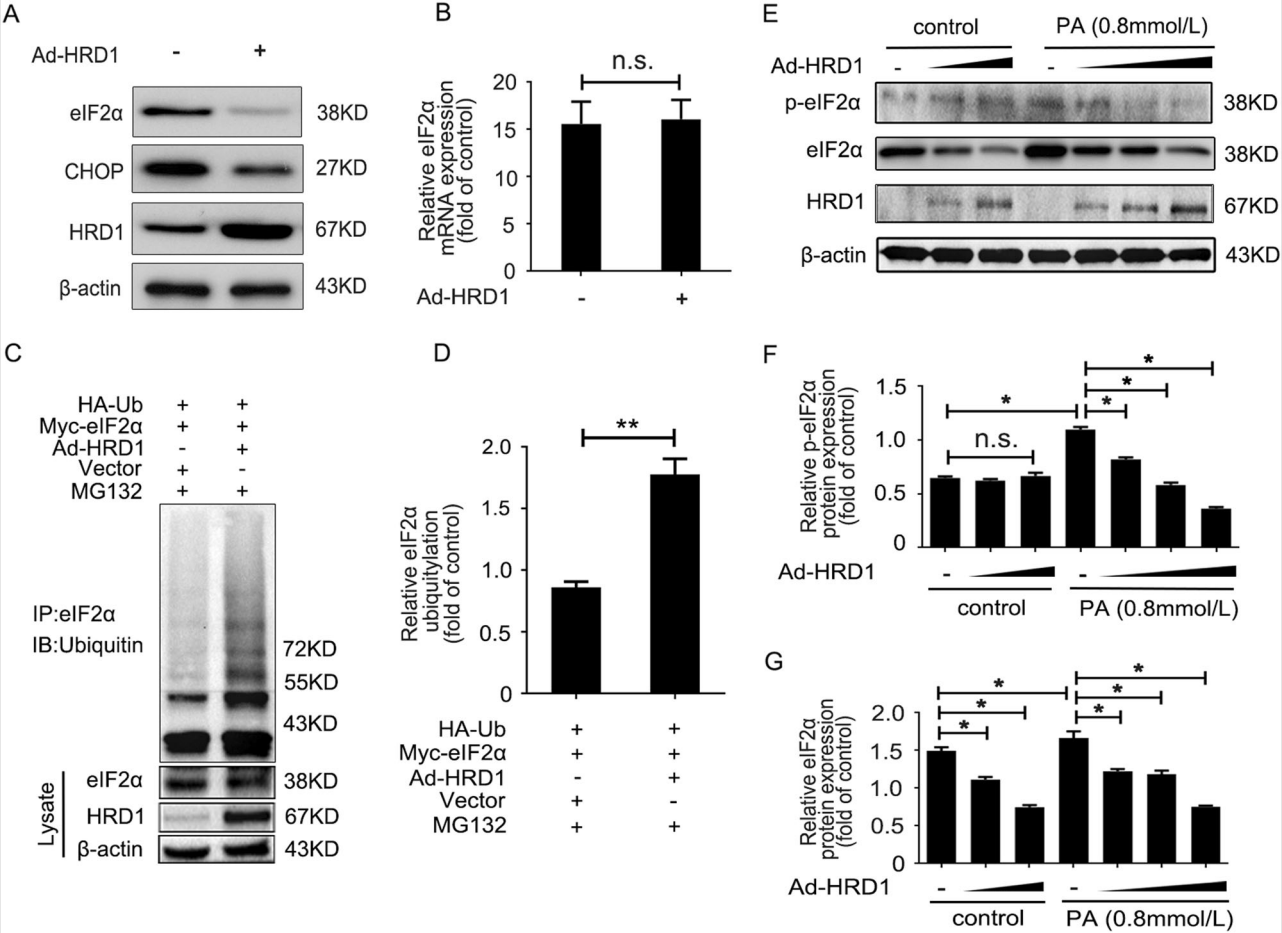
HRD1 promotes eIF2α ubiquitylation and degradation. **a** The endogenous eIF2α and CHOP expression was significantly decreased by HRD1 overexpression in HKC-8 cells. **b** The mRNA level of eIF2α showed no significant differences in the presence or absence of HRD1 overexpression. **c** More ubiquitin conjugated to eIF2α was detected in the cells overexpressing HRD1 compared with no HRD1 transfection. **d** Quantification of ubiquitin conjugated to eIF2α as in (**c**), normalized to actin expression. **e** Palmitic acid (PA) treatment of HKC-8 cells significantly increased the expressions of p-eIF2α and eIF2α, but their expressions were dose-dependently decreased with Ad-HRD1 infection. Quantification of p-eIF2α (**f**) and eIF2α (**g**) expression as in (**d**), normalized to actin expression. Bars are means ± S.E. from three independent experiments. n.s. means no significance, *P < 0.05, **P<0.01

The physiological significance of HRD1 in the regulation of endogenous eIF2α expression was evaluated by infecting HKC-8 cells with Ad-HRD1 for 24 h. As shown in Fig. 6a, the expressions of endogenous eIF2α and CHOP were significantly decreased by HRD1 overexpression in HKC-8 cells. However, the mRNA level of eIF2α showed no significant differences when HRD1 was overexpressed (Fig. 6b). These findings verified that HRD1 decreased eIF2α expression at the post-translational level.

We performed further investigations on the potential mechanism responsible for the effect of HRD1 on eIF2α expression using MG132, a proteasome inhibitor, to inhibit protein degradation. As shown in Fig. 6c and Supplementary Fig. 3, we found more than twice as much ubiquitin was conjugated to eIF2α in cells infected with Ad-HRD1 than transfected with vector only. The means of data for ubiquitin-conjugated eIF2α are shown in Fig. 6d. These results suggest that HRD1 serves as an E3 ligase that promotes eIF2α ubiquitylation and degradation by the proteasome.

HRD1 regulation of eIF2α in HKC-8 cells in an injured condition was examined by infecting HKC-8 cells with Ad-HRD1 for 24 h with or without treatment with PA (0.8 mmol/l) for 48 h. Western blots revealed that PA significantly increased the expressions of p-eIF2α and eIF2α, but their expressions were dose-dependently decreased with Ad-HRD1 infection (Fig. 6e). The means of data for the expressions of p-eIF2α and eIF2α are shown in Figs. 6f, g. These findings suggested that HRD1 prevents apoptosis in HKC-8 cells by mediating eIF2α degradation.

### eIF2α overexpression suppressed the HRD1 protection of HKC-8 cells against apoptosis

**Fig. 7:**
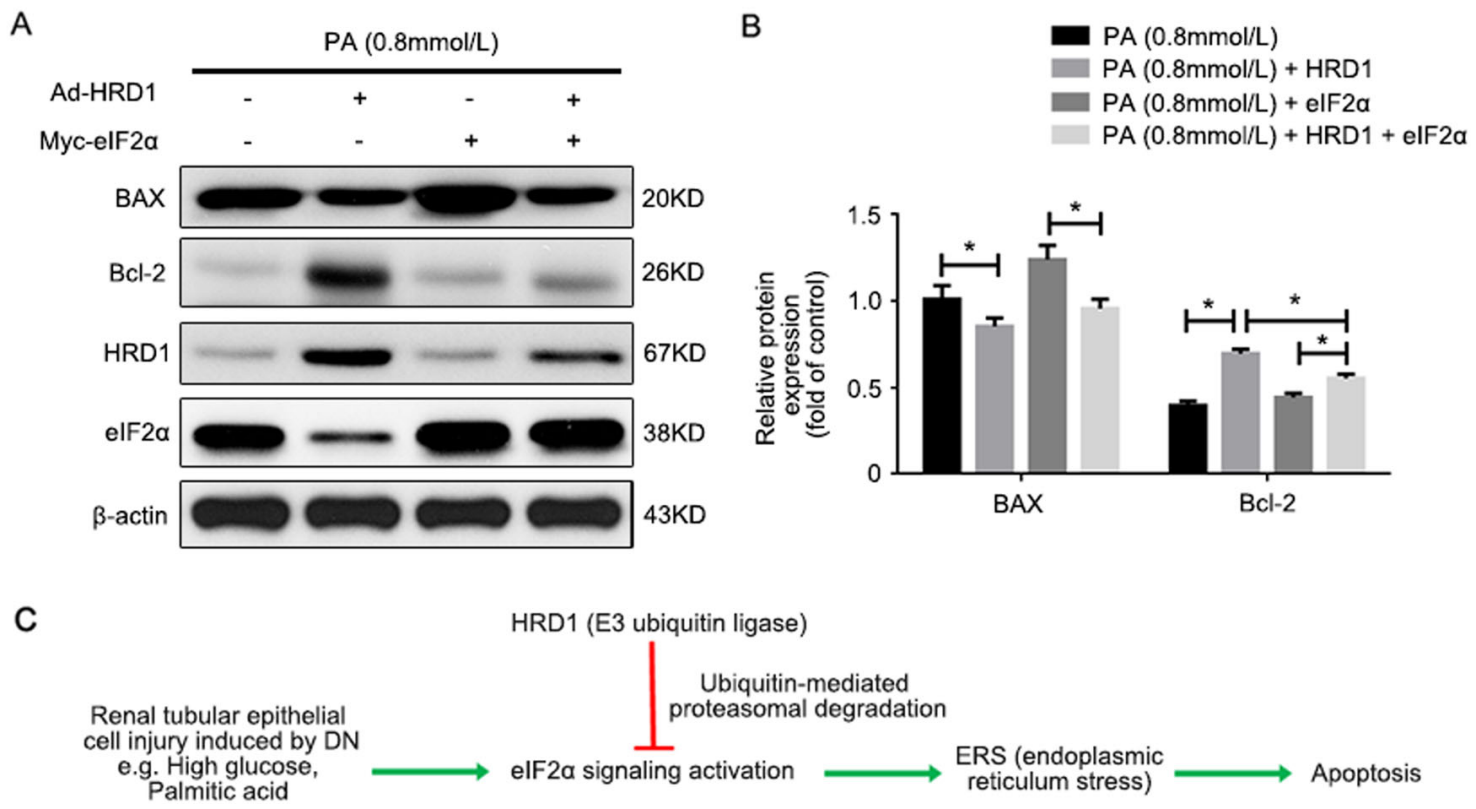
eIF2α overexpression suppressed the HRD1 protection of HKC-8 cells against apoptosis. **a** Treatment with palmitic acid (PA) alone and forced expression of eIF2α markedly increased BAX expression, but decreased Bcl-2 expression. Ad-HRD1 infection significantly suppressed BAX and restored Bcl-2 expression. However, the HRD1 protection induced by Ad-HRD1 infection in PA-treated HKC-8 cells was blunted by transfection with Myc-eIF2α. **b** Quantitation of western blot data for protein expressions as in (**a**), normalized to actin expression. Bars are means ± S.E. from three independent experiments. *P *<* 0.05. **c** Schematic diagram showing the promotion of ubiquitylation and degradation of eIF2α by HRD1 benefit renal apoptosis

The protective effect of HRD1 against apoptosis in HKC-8 cells induced by PA was caused by regulation of eIF2α ubiquitylation and degradation, as confirmed by transfecting eIF2α plasmids into HKC-8 cells treated with PA (0.8 mmol/l) for 48 h, and co-transfection with or without Ad-HRD1. Western blots performed to examine BAX and Bcl-2 expression revealed that PA treatment alone and forced expression of eIF2α markedly increased BAX expression, but decreased Bcl-2 expression. The Ad-HRD1 infection significantly suppressed BAX and restored Bcl-2 expression. However, HRD1 protection against Ad-HRD1 infection in PA-treated HKC-8 cells was blunted by transfection with Myc-eIF2α, as shown in Fig. 7a. The means of data for BAX and Bcl-2 expression under the variety conditions are shown in Fig. 7b. These data demonstrated that HRD1 suppressed tubular epithelial cell apoptosis by promoting eIF2α ubiquitylation and degradation.

## Discussion

Protein ubiquitylation is a sequential three-step enzymatic reaction consisting of ubiquitin activation by a single ubiquitin activating enzyme (E1), transfer to ubiquitin-conjugating enzymes (E2) that act as ubiquitin carrier proteins by forming a thioester bond with the E2-ubiquitin (E2-Ub) complex, and subsequent recruitment by substrate-ubiquitin E3 ligases and transfer of the activated ubiquitin linked to the target for its degradation by the ubiquitin-proteasome system (UPS)^22^.

Growing evidence now implicates the UPS in the pathogenesis of a variety of diseases, including kidney injury^12,23^. The activation of TGF-β, MAPK, and Nrf2, which are key signaling pathways related to renal fibrosis, was associated with degradation of some negative signaling proteins through the UPS. The degradation of negative protein Smad7 by ubiquitylation led to TGF-β activation^24^. IκB, a protein suppressed by NFκB and that plays a critical role in renal disease, was degraded by the UPS to allow nuclear translocation of NFκB for participation in gene transcription^25^. By contrast, degradation of other proteins would be beneficial in renal disease. Our previous study demonstrated that the protective effects of resveratrol on DN were associated with HRD1 upregulation and HRD1 promotion of IGF-1R ubiquitylation and degradation^19^. HRD1, as an E3 ubiquitin ligase, usually interacts with its specific substrate and then sends the target protein for degradation. Our LC-MS/MS screening of the HRD1-binding proteins in HKC-8 cells revealed 54 candidate peptide substrates for HRD1 ubiquitylation, and eIF2α was in the top 10. Therefore, the physical role of eIF2α ubiquitylation by HRD1 needs to be evaluated.

The eIF2α protein is a central player in the PERK-eIF2α-ATF4-CHOP signaling pathway associated with ER stress. Various renal injury factors, including HG and fatty acids, are known to induce oxidative and endoplasmic reticulum stress (ERS), and therefore activation of the UPR and apoptosis. In our present study, we observed an increased eIF2α expression and decreased HRD1 expression following induction of apoptosis in HKC-8 cells by PA and HG. HRD1 also showed a lower expression in DN kidney tissues than in control tissues. In addition, apoptosis was suppressed in HRD1-overexpressing HKC-8 cells. HRD1 overexpression also decreased the expression of eIF2α and p-eIF2α in HKC-8 cells. We reasoned that HRD1-mediated eIF2α ubiquitylation and degradation inhibited tubular epithelial cell apoptosis. We also found that eIF2α phosphorylation was decreased in HRD1-overexpressing HKC-8 cells and ubiquitylation of eIF2α was induced. The effect of protein ubiquitylation on phosphorylation needs further investigation in the future.

Previous research has also suggested that CHOP deficiency significantly decreases the expression of the renal fibrosis markers collagen I, fibronectin, α-smooth muscle actin, and plasminogen activator inhibitor-1 in the kidneys of a mouse unilateral ureteral obstruction (UUO) model, and CHOP knockout also ameliorated tubular apoptosis and inflammatory cell infiltration in the UUO kidneys^26^. CHOP is downstream of eIF2α in the PERK-eIF2α-ATF4-CHOP pathway of ER stress. The phosphorylation of eIF2α induced by PERK activation upregulated ATF4 and CHOP, thereby initiating cell apoptosis. We believed that CHOP expression would be downregulated if eIF2α phosphorylation and expression were inhibited, and this would prevent cell injury and death. The ubiquitylation and degradation of eIF2α regulated by HRD1 protein therefore might represent an important intervention target to prevent the progression of CKD.

In conclusion, HG and PA treatments induced tubular epithelial cell injury and apoptosis related to ER stress and activation of the PERK-eIF2α-ATF4-CHOP pathway. HRD1 promoted eIF2α ubiquitylation and degradation, thereby providing a protective mechanism that suppressed tubular epithelial cell apoptosis. These findings indicate that HRD1 expression is essential for amelioration of tubular cell injury and implicate HRD1 as a novel therapeutic target for renal disease.

## Materials and methods

### Reagents, antibodies, and plasmid constructs

Antibodies against Bcl-2, BAX, PARP, caspase-3, CHOP, Ubiquitin, eIF2α, and p-eIF2α were purchased from Cell Signaling Technology (Beverly, MA, USA). HRD1 antibody for western blot analysis and eIF2α antibody for immunofluorescence staining were obtained from Abcam (Cambridge, MA, USA). HRD1 antibody for immunohistochemistry (IHC) was purchased from Abgent (San Diego, CA, USA). Antibodies against β-actin, tubulin, and GAPDH were acquired from Proteintech (Chicago, IL, USA). The annexin V-FITC/PI staining kit was purchased from Invitrogen (Grand Island, NY, USA). The HA-Ub construct was the gift from Dr. Fei Sun (Wayne State University). Enhanced green fluorescent protein (EGFP)-tagged HRD1 adenovirus (Ad-HRD1) and Myc-eIF2α were obtained from Genechem (Shanghai, China).

### Cell cultures

Human renal tubular epithelial (HKC-8) cells were maintained in DMEM:F12 media supplemented with 10% fetal bovine serum and 100 units/ml penicillin. Cells were cultured in a standard humidified incubator at 37 °C in a 5% CO_2_/95% air atmosphere, and the culture media were changed every second day.

### Apoptosis assays

Apoptosis was determined by flow cytometry analysis (BD Biosciences, Heidelberg, Germany). HKC-8 cells subjected to different treatments were harvested, lysed in 0.25% cold trypsin without EDTA, washed with PBS, and stained with fluorescein isothiocyanate (FITC)-Annexin V and propidium iodide (PI) according to the manufacturer’s protocol (Annexin V-FITC Apoptosis Detection Kit, Vazyme, Nanjing, China). Apoptotic cells (Annexin V-positive and PI-negative) were then quantified by flow cytometry using the BD analysis program.

### Western blot analysis

The lysates of HKC-8 cells subjected to different treatments were separated by SDS-PAGE, transferred to PVDF transfer membranes (Millipore), and blocked with 5% nonfat milk. The proteins were probed with primary antibodies against HRD1 (1:1000), eIF2α (1:1000), p-eIF2α (1:1000), caspase-3 (1:1000), Bcl-2 (1:1000), Bax (1:1000), PARP (1:1000), CHOP (1:1000), followed by incubation with horseradish peroxidase-conjugated secondary antibodies.

### IHC and immunofluorescence of DN kidney tissues

Kidney tissues were taken from 12-week-old male *db/db* mice that had developed DN, while the kidney tissues from *db/m* mice served as the nondiabetic controls. Morphological changes in the kidney lesions of both groups of mice were determined by HE, PAS, and PASM staining. The kidney tissues were fixed overnight with 4% paraformaldehyde at 4 °C. The samples were dehydrated, embedded in paraffin, and sectioned into 3-µm-thick transverse sections. The sections were dewaxed, treated with 3% H_2_O_2_ for 15 min, microwaved for 15 min to unmask antigens, washed in phosphate-buffered saline containing Tween-20 (PBST), and then incubated with rabbit anti-HRD1 (1:100 dilution) overnight at 4 °C. After three washes with PBST, the sections were incubated with secondary antibody for 30 min at 37 °C. The sections were then rinsed, and diaminobenzidine was added as a chromogen.

For immunofluorescent staining, HKC-8 cells were fixed in 4% paraformaldehyde for 25 min at room temperature. Cells were then extensively washed three times with PBS to remove any debris and lysed with 0.1% Triton X-100 in PBS for 20 min. After three washes with PBS, the cells were blocked with 1% BSA for 1.5 h at 37 °C, and thereafter incubated with rabbit anti-HRD1 antibody and mouse anti-eIF2a antibody at 4 °C overnight. After three washes with PBS, the cells were incubated with the relevant secondary antibodies for 1 h at 37 °C. The cells were then stained with 4’,6-diamidino-2-phenylindole for 2 min and washed with PBS. All images were obtained using an Olympus confocal microscope and processed using Photoshop software.

### Co-immunoprecipitation

HKC-8 cells were harvested and lysed in cold lysis buffer (50 mM Tris, 150 mM NaCl, 1 mM EDTA, 0.5% (v/v) NP-40, 10% (v/v) glycerol, 1 mM Phenylmethanesulfonyl fluorid (PMSF), and a complete protease inhibitor cocktail tablet) and washed with 100 μl protein A/G agarose beads in 1 ml lysis buffer. The lysates were then incubated with anti-HRD1 antibody, anti-eIF2α antibody, or control IgG overnight with the protein A/G agarose beads. The complexes were washed three times with lysis buffer and resuspended in 2 × SDS loading buffer. The immunoprecipitated proteins were eluted from the beads by incubation at 95 °C for 5 min. The eluted proteins were detected by immunoblotting after separation by SDS-PAGE.

### Flow cytometry analysis

Apoptotic cells were evaluated by flow cytometry using an Annexin V-FITC/PI staining kit. After washing with cold PBS, the cells were resuspended in binding buffer (100 mmol/l HEPES, 100 mmol/l NaCl, and 25 mmol/l CaCl_2_ (pH 7.4)) and stained with Annexin V-FITC/PI at room temperature in darkness for 15 min. Apoptotic cells were then evaluated by gating PI and Annexin V-positive cells on an FACSCalibur instrument (BD Biosciences). All experiments were performed in triplicate.

### TUNEL assay

The HKC-8 cells subjected to different treatments were seeded on coverslips in fresh medium, grown overnight in 24-well plates, and then incubated for 24 h. TUNEL staining was performed with a TUNEL BrightRed Apoptosis Detection Kit (Vazyme, Nanjing, China) according to the manufacturer’s instructions. All images were obtained using an Olympus fluorescence microscope and processed using Photoshop software.

### RNA extraction, purification, and real-time PCR analyses

Total RNA was extracted using the TRIzol RNA isolation system (Invitrogen). The cDNA synthesis was performed using a First Strand cDNA synthesis kit (Roche, Basel, Switzerland), as described previously^27^. The mRNA was quantified by real-time PCR using a LightCycler 480 II Sequence Detection System (Roche). The following primers were used: HRD1, forward 5′-AACCCCTGGGACAACAAGG-3′ and reverse 5′-GCGAGACATGATGGCATCTG-3′; eIF2α, forward 5′-TTGAACTGTTGTGACCCCGAC-3′, reverse 5′-CGTAGTCTGCCCGATTTTGC-3′; β-actin as internal control, forward 5′-GCAAGTGCTTCTAGGCGGAC-3′ and reverse 5′-AAGAAAGGGTGTAAAACGCAGC-3′.

### Ubiquitylation assays

The eIF2α ubiquitylation assay was conducted by transfecting Myc-eIF2α, HRD1, HA-ubiquitin, and empty vector plasmids into HEK293 cells with Lipofectamine 2000 for 24 h. Cells were then treated with 20 µM of the proteasome inhibitor MG132 for 6 h, washed twice in ice-cold PBS, and then solubilized in RIPA lysis buffer. The lysates were centrifuged to obtain cytosolic proteins and incubated with anti-eIF2α antibody overnight and then with protein A/G agarose beads for a further 4 h at 4 °C. The beads were washed three times with lysis buffer and the proteins were released from the beads by boiling in 2 × SDS loading buffer. The released proteins were analyzed by immunoblotting with antiubiquitin antibody.

### Statistical analysis

Statistical analyses were performed using statistical analysis software SPSS 13.0. Data were expressed as the mean ± S.E. Analysis of variance was used to determine the statistical differences among the groups. A *p* value < 0.05 was considered statistically significant.

## Electronic supplementary material

Supplemental data
