# Supplementary material for: HRD1 prevents apoptosis in renal tubular epithelial cells by mediating eIF2α ubiquitylation and degradation
Source: Cell Death Dis. 2017 Dec 11;8(12):3202. doi: 10.1038/s41419-017-0002-y (PMC5870601; doi:10.1038/s41419-017-0002-y)

Supplemental Figure Legend

Supplemental Figure 1. Glucose and palmitic acid induces death of HKC-8 cells. (A) HKC-8 cells were treated with glucose (0, 10, 20, and 30 mmol/L) for 24h, and then the number of cell death was analyzed. (B) HKC-8 cells were treated with PA (0, 0.2, 0.4, 0.6 and 0.8 mmol/L) for 24h, and then the number of cell death was analyzed. The results indicated that either high glucose or PA induced renal tubular epithelial cell death in a dose-dependent manner. Bars are means ± S.E. from three independent experiments. ★★$P<0.001$

Supplemental Figure 2. HRD1 is downregulated in apoptotic HKC-8 cells.
HKC-8 cells were treated with glucose (0, 10, 20, and 30 mmol/L) for 24h. Glucose treatment inhibited HRD1 expression in a dose-dependent manner. The mass weight of HRD1 on western bot was around 67KD.

Supplemental Figure 3. HRD1 promotes eIF2α ubiquitylation and degradation.
More ubiquitin conjugated to eIF2α was detected in the cells overexpressing HRD1 compared with no HRD1 transfection, which demonstrated that HRD1, as an E3 ligase, promotes eIF2α ubiquitylation and degradation by the proteasome. There was a pencil line on the original blot membrane.

Supplemental Figure 1

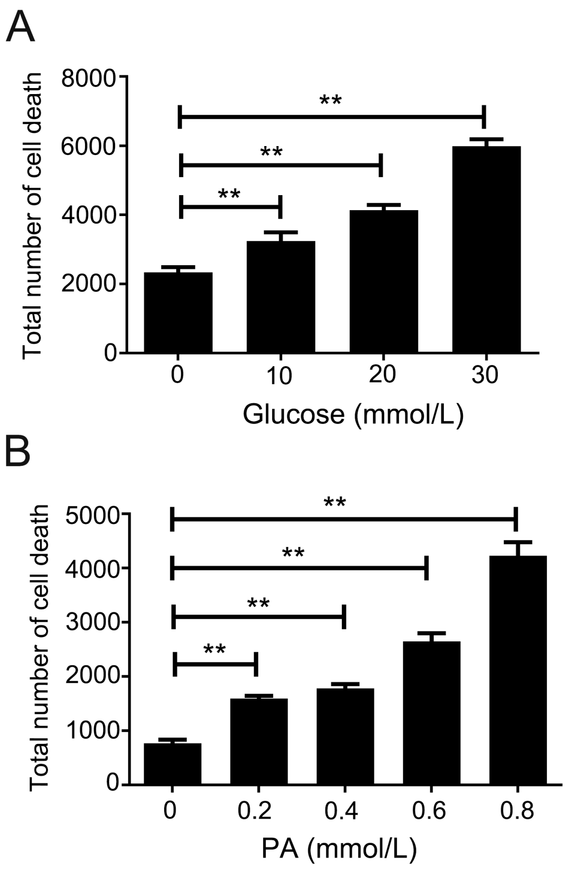

Supplemental Figure 2

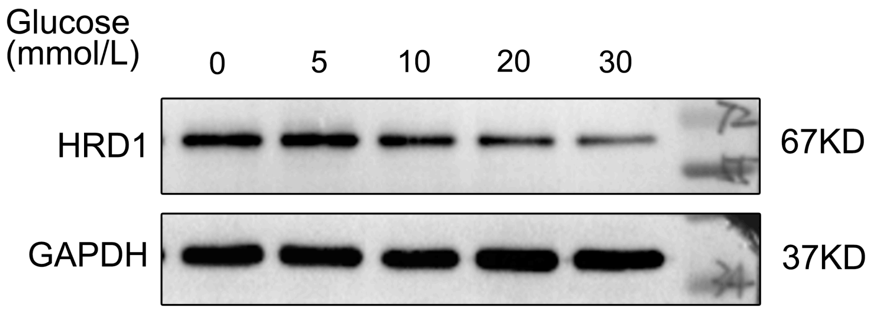

Supplemental Figure 3

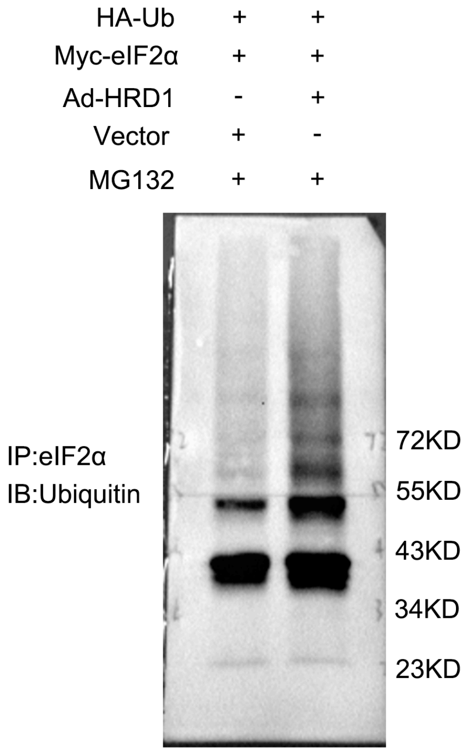

Supplement: Supplementary file 1 — Supplemental data [file 41419_2017_2_MOESM1_ESM.pdf]
